# Multilevel analysis of determinants of anemia among young women (15-24) in sub-Sahara Africa

**DOI:** 10.1371/journal.pone.0268129

**Published:** 2022-05-09

**Authors:** Misganaw Gebrie Worku, Tesfa Sewunet Alamneh, Achamyeleh Birhanu Teshale, Yigizie Yeshaw, Adugnaw Zeleke Alem, Hiwotie Getaneh Ayalew, Alemneh Mekuriaw Liyew, Zemenu Tadesse Tessema, Getayeneh Antehunegn Tesema

**Affiliations:** 1 Department of Human Anatomy, University of Gondar, College of Medicine and Health Science, School of Medicine, Gondar, Ethiopia; 2 Department of Epidemiology and Biostatistics, Institute of Public Health, College of Medicine and Health Sciences, University of Gondar, Gondar, Ethiopia; 3 Department of Human Physiology, University of Gondar, College of Medicine and Health Science, School of Medicine, Gondar, Ethiopia; 4 Department of midwifery, School of Nursing and Midwifery, College of Medicine and Health Sciences, Wollo University, Dessie, Ethiopia; University of Washington, UNITED STATES

## Abstract

**Background:**

Anemia is a disorder by which the body’s red blood cells are inadequate to fulfill The physiological needs of the body. The World Health Organization (WHO) defines anemia as having a hemoglobin (Hb) level of less than 120 g/l for nonpregnant women and 110 g/l for pregnant women. It has serious implications for human health as well as negative social and economic consequences like decreased workforce, impaired learning, and stunted child development. As these women are highly vulnerable to different micro and macro-nutritive deficiency associated with rapid physical, mental and psychological development, particular attention should be given to a young woman (15–24). Therefore this study assesses the magnitude and determinants of anemia among young women in sub-Saharan Africa (SSA).

**Methods:**

This was a secondary data analysis based on the Demographic and Health Surveys (DHS) data conducted in sub-Saharan Africa. We pooled the most recent DHS surveys done in 31 sub-Sahara Africa and a total weighted sample of 88, 832 young women (15–24 years) were included. At bivariable analysis, variables with a p-value of ≤0.2 were selected for multivariable analysis, and at the multivariable analysis variables with a p-value of ≤0.05 were considered as a significant factor associated with anemia among young women (15–24 years).

**Results:**

The pooled prevalence of anemia among young women (15–24) in sub-Sahara Africa was 42.17% [95%CI: 41.85, 42.50]. Young women of aged 20–24 years [AOR = 0.92, 95%CI: 0.89–0.95], women from rich household [AOR = 0.83, 95%CI: 0.80–0.87], young women with primary [AOR = 0.7, 95%CI: 0.67–0.72], secondary [AOR = 0.72, 95%CI: 0.69–0.75] and higher educational status [AOR = 0.58, 95%CI: 0.53–0.64], married women [AOR = 1.12, 95%CI: 1.08–1.17], divorced/separated/widowed women [AOR = 1.16, 95%CI: 1.08–1.25], women who use modern contraceptive [AOR = 0.65, 95%CI: 0.62–0.67], young women who ever had terminated pregnancy [AOR = 1.22, 95%CI: 1.14–1.29], overweight young woman [AOR = 0.79, 95%CI: 0.76–0.82] and young women from female-headed household [AOR = 0.94, 95%CI: 0.91–0.97] were the individual-level factors that significantly associated with anemia of young women. Meanwhile, being a rural dweller [AOR = 0.82, 95%CI: 0.79–0.85] and high community educational level [AOR = 0.87, 95%CI: 0.70–0.97] were the community level determinant of anemia. Interclass correlation coefficient (ICC), Median Odds Ratio (MOR) and Percentage change in variance (PCV) were done for the assessment of the random effect model of the multilevel analysis. The ICC value in the null model was 0.05, which indicates that 5% of the variation in anemia among young women in sub-Saharan Africa was attributed to community-level factors.

**Conclusion:**

The prevalence of anemia among young women in this study was higher compared with reports from the previous studies. Divorced/separated/widowed women, married women and women with ever terminated pregnancy, young women with primary, secondary and higher educational achievement, being rural dwellers, young women aged 20–24 years, being from rich households and women who used modern contraceptives were factors that significantly associated with anemia among young women. Therefore, particular attention should be given to those higher-risk women including, young women with a history of a terminated pregnancy, those from rural areas and young women aged 15–19 years to reduce the burden of anemia among these young women as the continuity of the future generation depends on the health of young women.

## Background

Anemia is a condition in which the body’s red blood cells are insufficient to meet its physiological need. Anemia affects 1.62 billion individuals worldwide, accounting for 24.8% of the population [1]. According to the World Health Organization (WHO) report, the prevalence of anemia among reproductive-age women is 57.1%, with the highest prevalence in Central (61%) and West Africa (61%) and the lowest in South Africa (34%) [2]. The World Health Organization (WHO) defines anemia as having a hemoglobin (Hb) level of less than 120 g/l for nonpregnant women and 110 g/l for pregnant women aged 15 years and above [3]. Anemia has serious implications for human health as well as long-term negative social and economic impacts [4]. The WHO estimates that nearly two billion people are anemic accounts for one million deaths per year of which three-quarters occur in low and middle-income countries [5]. Anemia is one of the most common nutritional deficiency diseases that affect more than a quarter of the world’s population [6–9]. It also impairs blood oxygen circulation by reducing the volume of red blood cells leads to negative consequences for maternal and child health [10]. It also results in stunted child development, impaired learning and decreased work efficiency [10, 11]. Anemia harms the health of women in reproductive age and pregnant women, under-five and preschool children, adolescents and young women [6, 9, 12–15]. Besides, the increased burden of anemia among adolescent and young women is associated with a period of rapid physical growth, reproductive maturation and cognitive transformations, which leads to higher demands for macro and micronutrients [16].

Anemia reduces worker productivity, particularly in developing countries where physical labor is the norm [17, 18]. Because of these negative effects, the WHO divides anemia level as it is not a public health problem (prevalence≤4.9%), mild public health concern (5 to 19.9%), moderate public health concern (20.0 to 39.9%) and extreme public health concern (prevalence ≥40.0%) depending on its impact on the community [7, 11 19, 20]. Different studies have identified that the age of the respondent, marital status, educational level, nutritional status, wealth status, source of drinking water and type of toilet facility were the factors that significantly associated with anemia among young women [12, 21–25]. Despite the implementation of control programmers including iron supplementation, deworming and insecticide-treated bed net distribution, anemia remains a major concern among young women in sub-Saharan Africa (SSA) [11] and with the current trends of anemia, it is difficult to achieve sustainable development goals of anemia (reduction of anemia of reproductive age woman by 50%) by 2030. As eradication of all forms of malnutrition is one of the main targets of SDGs and through the reduction of malnutrition anemia prevalence can be reduced. It is associated with an increased risk of death with poor cognitive abilities [7–9] and severe cases, lower aerobic exercise capacity and heart failure [21]. Even though studies are estimating the magnitude of anemia among reproductive-age women and children, the researcher gives less attention to the impact of anemia on the health of young women. As these women are highly vulnerable to different micro and macro-nutritive deficiency because of rapid physical, mental and psychological development, particular attention should be given to this population. Besides, some previously published works and reports indicated that the health impact and economic burden of anemia are persisting high in sub-Saharan Africa. Therefore this study assesses the magnitude and determinants of anemia among young women in sub-Saharan Africa.

## Methods

### Data source

**Fig 1:**
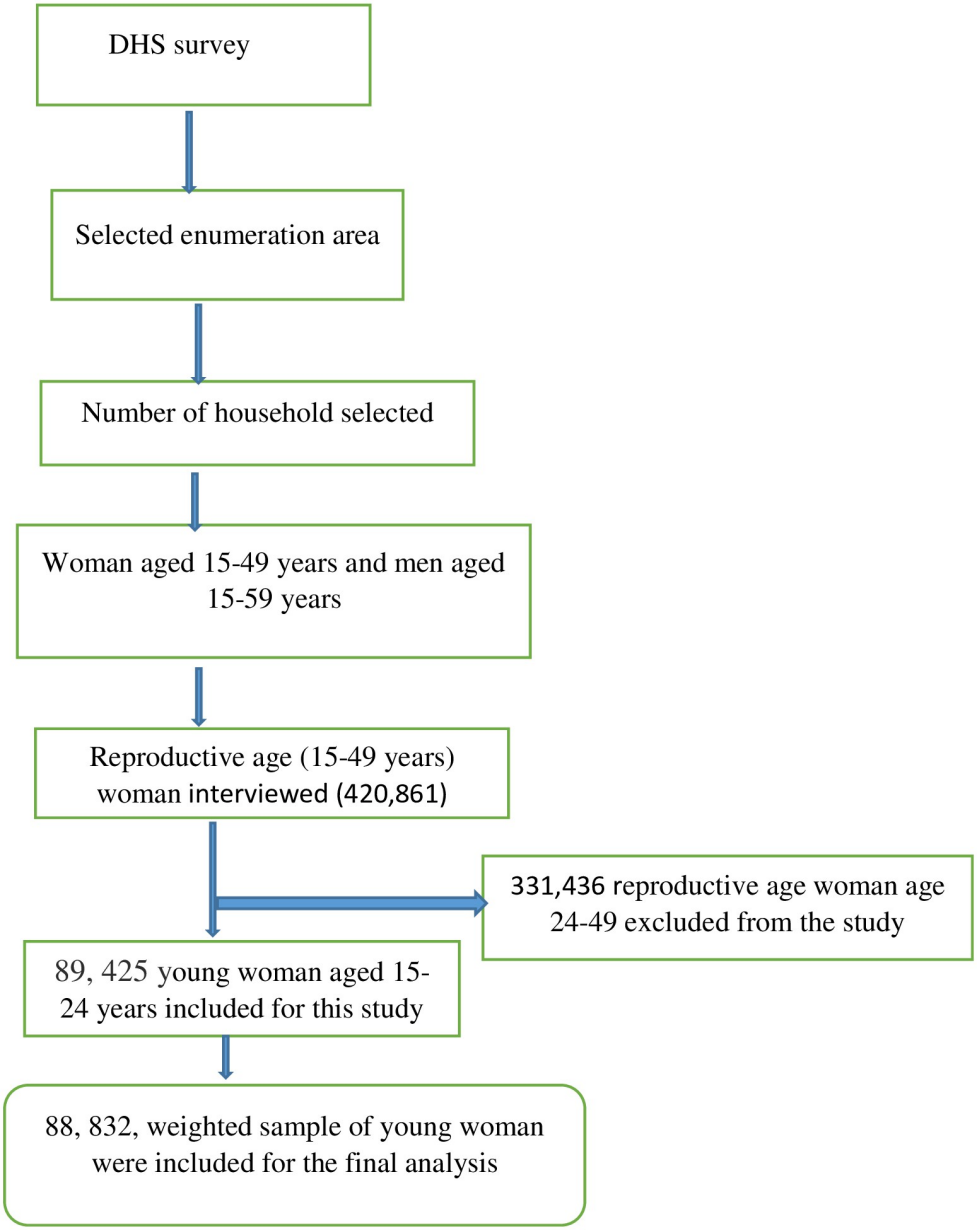
Flow diagram showing the sampling procedures of the DHS data.

A dataset from the most recent Demographic and Health Surveys (DHS) conducted in Sub-Saharan African countries (SSA) was used for this analysis. Young women aged 15–24 years from 31 SSA countries participating in the DHS that performed anemia testing were included. We pooled the most recent DHS surveys and a total weighted sample of 88, 832 young women (15–24 years) were included. The data from the DHS survey naturally form hierarchical nature of households within the cluster, household members within each household, interviewed women and men as a subset of household members and children of each interviewed woman. An enumeration area with a probability proportionate to the EA size was selected at the first stage and then a specified number of households per cluster were chosen at random from a newly produced household list with an equal probability at the second stage of sampling procedures. All women of 15–49 years and all men aged 15–59 years who were either permanent residents of selected households or visitors residing in the household the night before the survey were eligible for interview (Fig 1). From these national DHS survey data information on the basic health indicators including mortality, morbidity, family planning utilization, fertility, maternal and child health were collected. The survey year and total weighted sample included for this study were presented in Table 1.

**Table 1:** Survey year and total weighted sample included from each country.

	Country	Survey year	Weighted sample
East Africa	Ethiopia	2016	5796
	Burundi	2016/17	3554
	Tanzania	2015/16	5315
	Uganda	2016	2588
	Mozambique	2011	5456
	Zimbabwe	2015	3639
	Zambia	2018	5566
	Madagascar	2008	3274
	Rwanda	2014/15	2611
	Malawi	2015/16	3402
Central Africa	Cameroon	2018	2847
	Democratic Republic of Congo	2013/14	3806
	Congo	2011/12	2141
	Gabon	2012	2096
	São Tomée Príncipe	2008/9	972
West Africa	Benin	2017/18	3113
	Burkina Faso	2010	3266
	Ivory Coast	2011/12	1876
	Gambia	2013	1986
	Ghana	2014	1601
	Guinea	2018	2125
	Mali	2018	1871
	Niger	2012	1733
	Nigeria	2018	5006
	Senegal	2010/2011	2395
	Sierra Leone	2019	2827
	Togo	2013/14	1731
South Africa	Lesotho	2014	1393
	Namibia	2013	1716
	South Africa	2016	952
	Swaziland	2005	2161

### Variables of the study

#### Dependent variable

First, we dichotomized anemia in young women (15–24 years) into non-anemic which coded as “0” and anemic (by considering and merging mild, moderate, and severe anemia as anemic) which was coded as “1”. In the DHS data, women’s hemoglobin level was measured using HemoCue and the values were adjusted for altitude and smoking [26].

#### Independent variables

The independent variables were identified from different literature, which incorporates both the individual and community level variables. Age of respondent, educational level, marital status, wealth status, type of toilet facility, source of drinking water, body mass index (BMI), use of mosquito bed net, sex of household head, distance from the health facility, ever born children, modern contraceptive use, Cigarette smoking, ever terminated pregnancy and media exposure were the individual-level variables included. Whereas, residence, community poverty level and community educational level were community-level variables included for this study. Community poverty level and community educational level were created by aggregating their respective individual-level factor (wealth index) and (highest educational status), respectively. The median value was used to classify community poverty level as low (<50%) and high (≥50) as the variable was not normally distributed (Table 2).

**Table 2:** Description and measurement of independent variables.

Independent variables and their description/categorization	
Individual-level variables	
Age Group	The current age of the women and re-coded into two categories with values of “0” for 15–19, “1” for 20–24.
Wealth Index	The datasets contained a wealth index that was created using principal components analysis coded as “poorest”, “poorer”, “Middle”, “Richer”, and “Richest in the DHS data set.” For this study we recoded it in to three categories as “poor” (includes the poorest and the poorer categories), “middle”, and “rich” (includes the richer and the richest categories)
Distance to the health facility	Re-coded in two categories with a value of “0” if the woman perceived as it is not a big problem to get a medical help from the health facility and “1” if the woman perceived as it is a big problem to get a medical help from the nearby health facility.
Media exposure	A composite variable obtained by combining whether a respondent reads newspaper/magazine, listen to radio, and watch television with a value of “0” if a women were not exposed to at least one of the three medias, and “1” if a woman has access/exposure to at least one of the three medias.
Educational status	This is the minimum educational level a woman achieved and re-coded in to three groups with a value of “0” for no education, “1” for primary education, and “2” for secondary and above (combining secondary and higher education categories together).
Marital status	This was the current marital status of women and recoded in two categories with a value of “0” for unmarried (includes those who were never in union, divorced, widowed, and separated), and “1” for “married” (includes those living with partner and those who are married)
Sex of household	The variable sex of household head was recorded as male and female in the dataset and we used without change.
Cigarate smoking	The variable Cigarate smoking was recoded as “0” for woman who never smoke Cigarate and “1” for woman who smokes Cigarate.
Body mass index	The variable body mass index was recoded as “0” for women of BMI 18.5–24.5 (Normal), “1” for women of BMI <18.5 (underweight) and “2”for woman with BMI of >24.5 (overweight).
Modern Contraceptive usage	Recoded in to two categories with value of 0 for “no” if a women don’t use any of the modern contraceptive methods, and 1 for “Yes” if a women use any of the modern contraceptive methods. of either of or combination of the following methods (female sterilization, implant, intrauterine device (IUD), injectable, oral contraceptive, emergency contraceptive, condom, lactational amenorrhea and periodic abstinence)
Ever terminated pregnancy	The variable ever terminated pregnancy was categorized in to two categories as “0” for woman who have no any form of terminated pregnancy and “1” for woman have ever terminated pregnancy.
Toilet facility	The variable toilet facility was categorized in to two categories “0” which stands from unimproved toilet facility flush to somewhere else, flush don’t know where, pit latrine-without slab/open pit, no facility/bush/field, bucket toilet, hanging toilet/latrine and others) and “1” for improved toilet facility (flush-to piped sewer system, flush-to septic tank, pit latrine, unspecified, ventilated improved pit (VIP), pit latrine-with slab and composite toilet)
Source of drinking water	Source of drinking water was categorized as “0” for unimproved (unprotected well, unprotected spring, surface water, tanker truck, car with small tank, bottled water and other) and “1” for those who have improved water sources (piped in to dwelling, piped to yard/plot, pubic tab/stand pip, protected well, protected spring, rain water and bottled water).
Total children ever born	The variable total children ever born was categorized in to three categories “0” for those who have no child, “1” for those woman with 1–5 children and “2” for woman with more than 5 children.
Community level variables	
Community of poverty level	Measured by proportion of households in the poor (combination of poorer and poorest) wealth quintile derived from data on wealth index. Then it was categorized based on national median value as: low (communities in which <50% of women had poor socioeconomic status) and high (communities in which ≥50% of women had poor socioeconomic status) poverty level.
Community educational level	Measured by the proportion of educated women (combination of primary, secondary and higher education). It was categorized based on national media value as: low (community in which < 50% of women had no education) and high (community with ≥ 50% of women had educational attainment).
Type of place of residence	The variable place of residence recorded as rural and urban in the dataset was used without change.

### Data management and analysis

Stata version 14 software was used for data extraction, recoding and analysis. The data were weighted before any statistical analysis to restore the representativeness of the data and to get a reliable estimate and standard error. Descriptive statistics were done using frequencies and percentages. For this analysis we cannot use the standard logistic regression analysis as the hierarchal nature of the DHS data violates the independent assumptions of the standard binary logistic regression model and because of this, we fitted a multilevel mixed-effects generalized linear model (with the assumption that individual specific-effect are uncorrelated with the independent variables). At first, we were considering both the individual level, household level, and community level analysis. But when we see the analysis result there is no significant clustering effect at the household level and for the above-mentioned reason, we were going to use the two-level i.e. the individual and community level analysis. To balance the size of clusters and detect the random effect efficiently, we excluded an EA with less than 10 observation per cluster and the maximum number of individual observed were 213 per cluster. While conducting a multilevel logistic regression analysis four models; the null model containing only the outcome variable, a model I and II containing individual and community level variables, respectively, and model III, which contains both individual and community level variables were fitted. Intraclass Correlation Coefficient, Median Odds Ratio and Percentage Change in Variance were checked to assess the clustering effect. Since these models were nested, we used deviance to check the model comparison and the model with the lowest deviance were chosen. Both bivariable and multivariable multilevel logistic regression was done and variables with a p-value of ≤0.2 in the bivariable analysis were considered for multivariable analysis. Finally, variables with P-value ≤0.05 in the multivariable analysis were identified as significant factors associated with anemia among young women (15–24 years).

## Results

### Sociodemographic characteristic of study participants

A weighted sample of 88,832 young women aged 15–24 years were included for this study. More than half (53.62%) of the participants were aged 15–19 years and about 45.99% of them were from rich households. Considering educational status, nearly 45% of the participants had achieved secondary level education and the majority (83.72%) of the participants didn’t use modern contraceptives. The majority (72.52%) of the participants had been exposed to different media and more than half (53.01%) of them had access to an improved toilet facility. The majority (61.17%) of the participants were from rural dwellers and about 97.41% of them were from the community of higher educational level (Table 3).

**Table 3:** Sociodemographic characteristics of study participants (N = 88, 832).

Variable		Frequency	Percentage
Respondent age	15–19	47629	53.62
	20–24	41203	46.38
Wealth status	Poor	30931	34.82
	Middle	17052	19.20
	Rich	40850	45.99
Educational status	No	15986	18.00
	Primary	30623	34.47
	Secondary	39435	44.39
	Higher	2788	3.14
Distance to health facility	Big problem	32580	36.68
	Not big problem	56252	63.32
Modern contraceptive use	No	74371	83.72
	Yes	14461	16.28
Marital status	Unmarried	50730	57.11
	Married	34520	38.86
	Divorced/separated/ divorced	3583	4.03
Ever terminated pregnancy	No	83788	94.32
	Yes	5044	5.68
Cigarette smoking	No	88376	99.49
	Yes	456	0.51
Body mass index	Normal	59734	67.24
	Underweight	11950	13.45
	Overweight	17149	19.30
Media exposure	No	24410	27.48
	Yes	64423	72.52
Sex of house hold head	Male	63318	71.28
	Female	25514	28.72
Toilet facility	Improved	47094	53.01
	Unimproved	41738	46.99
Source of drinking water	Unimproved	41273	46.46
	Improved	47559	53.54
Total children ever born	No	51600	58.09
	1–5	37183	41.86
	>5	49.60	0.06
Residence	Urban	34497	38.83
	Rural	54335	61.17
Community level education	Low	86535	97.41
	High	2300	2.59
Community poverty level	Low	44013	49.55
	High	44819	50.45

### Prevalence of anemia among young women in sub-Saharan Africa

**Fig 2:**
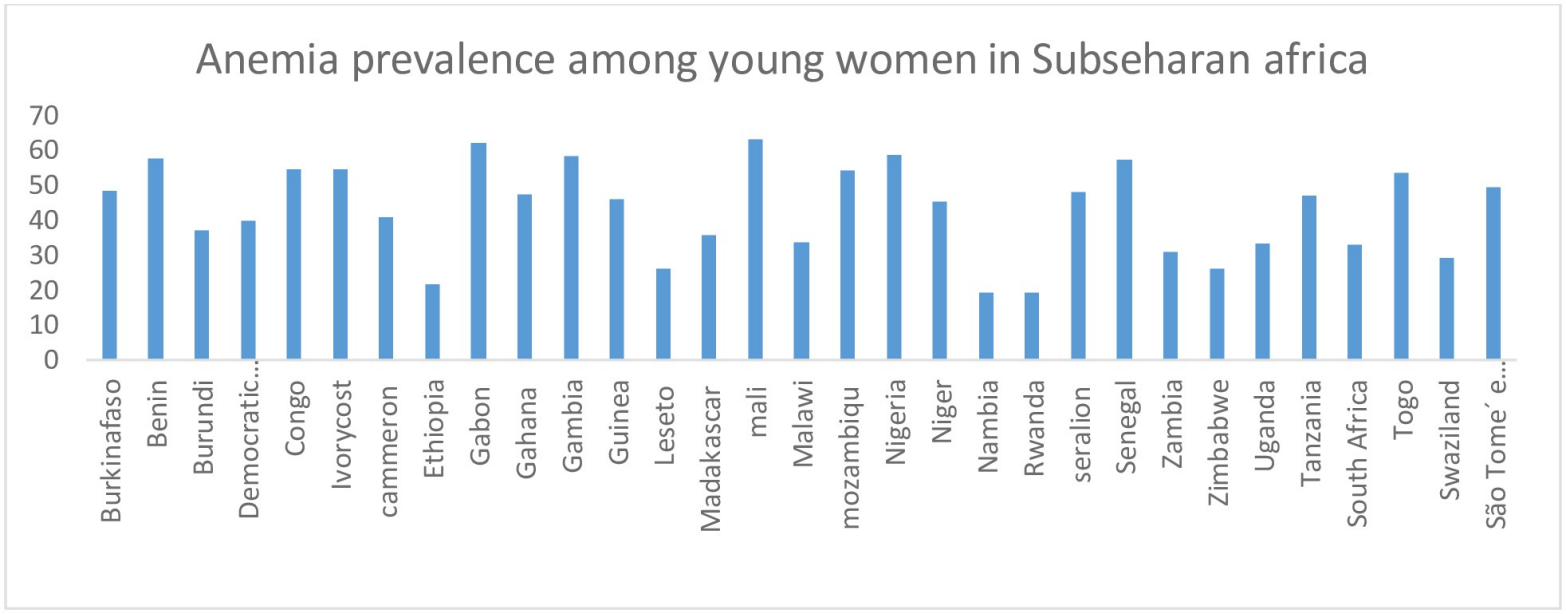
Showing the weighted prevalence of anemia among young women in sub-Sahara Africa.

The pooled weighted prevalence of anemia among young women (15–24) in sub-Sahara Africa was 42.17% [95%CI: 41.85, 42.50] ranged from 21.71% in Ethiopia to 62.95% in Mali (Fig 2).

### Random effect analysis

Interclass correlation coefficient (ICC), MOR and PCV were done to assess the random effect model of the multilevel analysis. The ICC value in the null model was 0.05, which indicates that community-level factors were attributed to 5% of the variation in anemia among young women in sub-Sahara Africa. Also, the highest MOR value 1.13 in the null model indicated that there is a higher clustering of anemia among young women. The percentage change in variance (PCV) in the final model, which is 42% indicated that the individual and community level factors together explained about 42% of the variation in anemia among young women (Table 4).

**Table 4:** Random effect model and model fitness for the assessment of anemia among young women in sub-Saharan Africa.

Parameter	Null model	Model I	Model II	Model III
Community-level variance	0.015343	.0152306	.0143298	.0135648
ICC	0.05	0.046	.0433	.0411
MOR	1.13	1.23	1.22	1.21
PCV	Reff	0.073	0.066	0.42
**Model fitness**				
Log likelihood	-60867.956	-60057.849	-60775.791	-59894.816
Deviance	121734.812	12015.698	121551.582	119789.632

### Determinants of anemia among young women in sub-Saharan Africa

In fitting of the final model age of the respondent, educational status, ever terminated pregnancy, nutritional status, current marital status, modern contraceptive use, household wealth status, type of toilet facility, source of drinking water, total children ever born, sex of household head, community-level education and residence were the significant determinant of anemia among young women. Young women aged 20–24 years had 8% [AOR = 0.92, 95%CI: 0.89–0.95) lower risk of being anemic compared with a woman aged 15–19 years. Women from rich households had 0.83 [AOR = 0.83, 95%CI: 0.80–0.87] times lower odds of developing anemia compared with young women from poor households. Looking at educational status, young women with primary [AOR = 0.7, 95%CI: 0.67–0.72], secondary [AOR = 0.72, 95%CI: 0.69–0.75] and higher educational status [AOR = 0.58, 95%CI: 0.53–0.64] had lower odds of anemia compared with uneducated young women. Married women had 1.12 [AOR = 1.12, 95%CI: 1.08–1.17] times higher odds of developing anemia compared with unmarried women. Similarly, divorced/separated/widowed women had 1.16[AOR = 1.16, 95%CI: 1.08–1.25] times higher odds of anemia compared with unmarried women. Interestingly, women who use modern contraceptives had 0.65[AOR = 0.65, 95%CI: 0.62–0.67] times lower odds of developing anemia compared with a young woman who did not use modern contraceptives. Young women who ever had terminated pregnancy had a 1.22[AOR = 1.22, 95%CI: 1.14–1.29] times higher risk of developing anemia compared with a woman with no history of pregnancy terminated. Overweighed young women had a 21% [AOR = 0.79, 95%CI: 0.76–0.82] lower chance of developing anemia compared with women with normal body mass index. Female-headed households had 0.94[AOR = 0.94, 95%CI: 0.91–0.97] times lower odds of developing anemia compared with male-headed households. Young women with total children of 1 to 5 [AOR = 1.09, 95%CI: 1.04–1.14] and greater than 5 [AOR = 2.20, 95%CI: 1.28–3.78] had higher odds of having anemia compared with women with no children. Rural dweller young women had 18% [AOR = 0.82, 95%CI: 0.79–0.85] lower risk of developing anemia compared with urban young women. Considering community-level education, women from the community of higher educational status had 0.87[AOR = 0.87, 95%CI: 0.70–0.97] times lower odds of being anemic compared with women from the community of low educational level (Table 5).

**Table 5:** Multilevel analysis for determinants of anemia among young women (15–24) in sub-Saharan Africa.

Variables		Anemia status		COR (95%CI)	AOR (95%CI)
		Non-anemic	Anemic		
Age	15–19	27408	23959	1	1
	20–24	20221	17244	0.96(0.94, 0.99)	0.92(0.89, 0.95)*
Marital status	Unmarried	30264	20466	1	1
	Married	19106	15413	1.21(1.17, 1.24)	1.12(1.08, 1.17)*
	Divorced/widowed/separate	1997	1584	1.15(1.08, 1.24)	1.16(1.08, 1.25)*
Educational status	No education	7802	8184	1	1
	Primary education	18263	12360	0.63(0.61, 0.66)	0.7(0.67, 0.72)*
	Secondary education	23499	15936	0.62(0.60, 0.65)	0.72(0.69, 0.75)*
	Higher education	1803	985	0.46(0.43, 0.51)	0.58(0,53, 0.64)*
Wealth status	Poor	17253	13678	1	1
	Middle	9619	7402	0.93(0.90, 0.95)	0.97(0.93, 1.01)
	Rich	24465	16385	0.79(0.77, 0.82)	0.83(0.80, 0.87)*
Distance to health facility	Big problem	18510	14070	1	1
	Not big problem	32857	23395	0.91(0.88, 0.93)	0.97(0.94, 1.00)
Modern contraceptive use	No	41750	32621	1	1
	Yes	9617	4844	0.63(0.60, 0.65)	0.65(0.62, 0.67)*
Ever terminated pregnancy	No	48706	35082	1	1
	Yes	2661	2383	1.26(1.19, 1.33)	1.22(1.14, 1.29)*
Body mass index	Normal	6642	5307	1	1
	Underweight	33842	25891	1.03(0.99, 1.08)	1.02(0.98, 1.06)
	Overweight	10883	6266	0.74(0.71, 0.76)	0.79(0.76, 0.82)
Cigarette smoking	No	51096	37280	1	1
	Yes	271	185	0.95(0.78, 1.16)	0.97(0.79, 1.18)
Toilet facility	Improved	27780	19295	1	1
	Unimproved	23568	18170	1.17(1.14, 1.20)	(1.01, 1.07)*
Current breast feed	No	39772	28409	1	1
	Yes	11595	9056	1.10(1.07, 1.14)	0.97(0.93, 1.01)
Media exposure	No	13949	10461	1	1
	Yes	37419	27004	0.91(0.88, 0.93)	1.03(0.99, 1.06)
Sex of house hold head	Male	36115	27203	1	1
	Female	15252	10262	0.88(0.85, 0.89)	0.94(0.91, 0.97)*
Source of drinking water	Unimproved	23329	17944	1	1
	Improved	28038	19522	0.87(0.85, 0.89)	0.97 (0.94, 1.01)
Total children ever born	No	30309	21291	1	1
	1–5	21042	16142	1.10(1.07, 1.13)	1.09(1.04, 1.14)*
	>5	17	33	2.66(1.56, 4.54)	2.20(1.28, 3.78)*
Community educational level	Low	50344	36188	1	1
	High	1023	1277	1.75(1.61, 1.91)	0.87(0.70, 0.97)*
Community poverty level	Low	25593	18420	1	1
	High	25774	19045	1.01(0.97, 1.04)	1.01(0.967, 1.03)
Residence	Urban	19537	14960	1	1
	Rural	31830	22505	1.04(1.02, 1.07)	0.82(0.79, 0.85)*

*P-value≤0.05

## Discussion

The prevalence of anemia among young women in sub-Sahara Africa was 42.17% [95%CI: 41.85%, 42.50%]. The finding of this study indicated that anemia is a major public health problem among young women according to the WHO classification of anemia for public health significance. Anemia prevalence among young women was higher than the studies conducted in Ethiopia, East Africa, Rwanda, Nepal and Chinese [12, 25, 27–33]. Meanwhile, the pooled prevalence of anemia in this study was lower than the prevalence reported in Ghana [34], Cameron [34], Congo democratic republic [34], Burkina Faso [34] and Asia [22]. This might be attributed to differences in the study settings and wider participants’ age group as we included only young women (15–24 years) [21]. In addition, these variations might be due to differences in cutoffs value used in the different organizations which are used to define anemia, geographic location as well as time differences [35].

The difference in the prevalence of anemia might also be due to the differences in socio-demographic characteristics, culture and adolescents’ behavioral and feeding habits or practices [33]. In the multilevel analysis age of respondent, educational status, ever terminated pregnancy, nutritional status, current marital status, modern contraceptive use, household wealth status, type of toilet facility, source of drinking water, total children ever born, sex of household head, community-level education and residence were the significant determinant of anemia among young women. Young women aged 20–24 years had lower odds of being anemic compared with their counterparts. This finding was supported by the study conducted in Asia [22]. A higher prevalence of anemia among early younger women could be due to the adverse effect of lower dietary iron intake and additional demand for iron imposed by iron loss during menstruation, pregnancy and lactation [13]. Because it is a phase of rapid growth, proper nutrition is essential for achieving full growth potential and failure to acquire optimal nutrition may result in delayed and stunted child growth as well as impaired organ remodeling. Because young women need additional micro and macronutrient, they are in danger of developing a variety of health problems, including anemia, if they do not receive appropriate nutrition [36].

Women who used modern contraceptives had lower odds of developing anemia supported by studies conducted in Ethiopia and Pennsylvania [24, 25, 31]. This might be associated with hormonal contraceptives are important to reduce excessive bleeding during the menstrual period that ultimately reduces blood loss over time [37, 38]. Young married women had a higher risk of developing anemia compared with unmarried women supported by previous studies conducted in Ethiopia [24, 25]. This might be due to as married women can get repeated pregnancy and this frequent pregnancy might lead to increased risk of hemorrhage before, during and after delivery that exposes them to a higher risk of anemia [39]. Women from rich households had lower odds of developing anemia compared with women from poor households. This finding was supported by the reports in Eastern Ethiopia [21] and Asia [22]. Young women with a primary and higher level of education had a lower risk of developing anemia compared with uneducated women, which was supported by other studies conducted in Ethiopia [12, 28] and Asia [22]. This is because young women who have some level of formal education can be aware of anemia during their life and take some preventive measures like eating iron-rich food and taking iron tablets during pregnancy [28]. This might also be associated with as educated females utilize adequate medical care and have good knowledge about appropriate nutrition and personal hygiene, which might be directly related to the risk of developing anemia [40]. Educated young women consume iron-rich food, utilize adequate healthcare facilities and manage a hygienic household environment, which is associated with a reduction in anemia among young women [41]. Young women with improved water sources have lower risk of developing anemia compared with a woman with unimproved water sources. This finding was in agreement with another study done in Japan [27] and Ethiopia [25]. Similarly, the likelihood of having anemia was greater among women with unimproved toilet facilities compared with a woman with improved toilet facility. This finding was supported by another studies conducted in Ethiopia and LaoPDR [25, 27].

This might be associated with the probability of environmental contamination of drinking water with parasites that causes anemia increases because of the inaccessibility of appropriate toilet facilities, which might, in turn, increase the risk of anemia [27].

In the present studies, women with ever terminated pregnancies had a higher risk of developing anemia compared with their counterparts. The finding is in agreement with another study [27]. In this study women who had ever children born of greater than five were more likely to develop anemia compared with women with no children, which was supported by a study conducted in Japan [27]. This might be associated with the physiological stress of several pregnancies, along with low-quality meals, parasites and poor sanitation poses significant hazards to mothers and their children. Being rural dwellers had a lower risk of developing anemia compared to the urban dweller, which was supported by other studies conducted in India [42], Malawi [43], and sub-Sahara Africa [44]. This might be because traditional staple foods consumed in rural households are more diversified and rich in iron content compared to those food taken by most urban dwellers and such diversified food patterns might have a positive role in meeting the adequacy of iron requirements for the young women [42]. Considering the nutritional status, overweight young women had a lower odds of developing anemia compared to women with normal nutritional statues, which is supported by studies conducted in China [32]. This might because as food insecurity is the major problem in developing countries leads to food deficiency and poor quality of diet, which finally end up with different health problems including anemia.

As anemia of young women is fatal because of rapid physical and mental growth and menstruation-associated risks, policymakers should give particular attention to this public health problem by taking into consideration the variation of anemia among different clusters by using the random effect analysis. Since the random effect analysis of anemia among young women indicated that there is a significant clustering in anemia and those woman in higher risk clusters have a higher risk of developing anemia which is only addressed by the random effect analysis of multi-level model that provides suitable epidemiological information on area-level variance and clustering. The multilevel random effect analysis implied to this study identified anemia high and low-risk community which is important to policy and decision-maker to give priority to this high-risk group to take possible intervention strategies early. Also, the random effect analysis estimate of anemia of young women showed that there is a variation in the occurrence of anemia among young women in different randomly selected clusters/communities, therefore, the estimate of random effect analysis is very crucial to identify a young woman who lived in higher anemia risk cluster and to give priority to that higher risk population group.

### Strength and limitation of the study

Since this study was based on weighted large representative data the statistical power of the study is high. We also use an appropriate statistical approach to accommodate the hierarchical nature of the data and also the unobserved heterogeneity of this study is accounted as we used random parameter approaches. This study had limitations in that the DHS survey was based on respondents’ self-report and might have the possibility of recall bias. In addition as this study was based on cross-sectional collected DHS data, we are unable to show the temporal relationship between anemia among young women and independent variables.

## Conclusion

The prevalence of anemia among young women in sub-Sahara Africa was higher than reports in previous studies. In this study age of the respondent, educational status, ever terminated pregnancy, nutritional status, current marital status, modern contraceptive use, household wealth status, type of toilet facility, source of drinking water, total children ever born, sex of household head, community-level education and residence were the significant determinant of anemia among young women in SSA. Therefore, program planners should focus on the provision of iron, folate and other micronutrients through different intervention programs including supplementation and fortification to reduce these devastating nutritional-related high prevalence of anemia. In addition, there should be improved water access and improved sanitation including toilet facilities to reduce the anemia burden among young women in SSA. Besides, particular attention should be given to those higher-risk women including, a young woman with a history of terminated pregnancy, those from poor economic status, and low educational achievement to reduce the burden of anemia among these young women for the betterment of the continuity of generations.

## Abbreviations

CI: Confidence Interval
CSA: Central Statistical Agency
DHS: Demographic Health Survey
EA: Enumeration Area
GDP: Gross Domestic Product
ICC: Intraclass Correlation Coefficient
LLR: Likelihood Ratio
LMIC: low and middle-income country
PCV: Proportional change in Variance
SSA: sub-Saharan Africa
WHO: World Health Organization
